# Attractiveness of Cattle Dung to Coprophilous Beetles (Coleoptera: Scarabaeoidea and Sphaeridiinae) and Their Segregation During the Initial Stages of the Heterotrophic Succession on a Pasture in Southeast Michigan

**DOI:** 10.1093/jisesa/ieaa040

**Published:** 2020-06-05

**Authors:** Thomas Wassmer

**Affiliations:** Biology Department, Siena Heights University, Adrian, MI

**Keywords:** heterotrophic succession, segregation, Aphodiinae, Scarabaeinae, Hydrophilidae

## Abstract

Only a few mostly older studies analyzed the heterotrophic succession of dung beetles in the Midwestern United States. Such studies are needed to track the impacts of the climate crisis on heterotrophic succession and the associated decomposition processes that are central to soil fertility and carbon sequestration. The current study closes this knowledge gap and provides an easy and efficient method to estimate the relative attractiveness of individual dung pads during heterotrophic succession. The dung beetle community of Carpenter Farm in Adrian, Southeast Michigan was sampled for an entire year, including the winter months, using 15 pitfall traps baited with fresh cow manure. Samples were collected after 48 h and again after 72 h exposure time from the bucket content while leaving the bait unhampered. Eighty-four percent of all beetles were caught in the early sample, but only 6 species were missing in the later sample. A cluster analysis based on Pianka’s niche overlap identified a statistically higher mean overlap than expected by chance in a null model (model RA3) and divided the species community clearly into three clusters separating most relocators from most dwellers. Despite using a different method, my results confirmed the successional position of most previously described species and added data for several species with poor or unknown successional state. The successional segregation between dwellers and relocators discovered by the cluster analysis was paralleled by a significantly larger body size of relocators across taxonomic groups as compared to dwellers.

Dung is the most important return of energy and nutrients consumed by animals into the food webs and nutrient cycles of ecosystems and the Earth’s biosphere (Doughty et al. 2016). The extent and quality of this return depend largely on the life processes of coprophilous organisms that aerate, breakdown, and digest dung and incorporate nutrients into the organic soil horizon (Piccini et al. 2018). Among the astonishing diversity of coprophilous organisms, coprophagous beetles stand out due to their abundance, trophic diversity, and variety of life cycles (Hanski and Cambefort 1991, Floate 2011). They are comprised of beetles from the superfamily Scarabaeoidea containing the family of Scarabaeidae with the subfamilies Scarabaeinae and Aphodiinae and the family Geotrupidae, and beetles from the subfamily Sphaeridiinae within the family Hydrophilidae. Whereas Scarabaeoidea are coprophagous both as larvae and adults, Sphaeridiinae are only coprophagous as adults while their dung dwelling larvae are carnivorous predators (Archangelsky 1997) adding to the ecological and trophic complexity of the dung ecosystem. A second major ecosystem service performed by coprophilous beetles reduces the survival of coprophagous Diptera and helminths that would otherwise flourish in feces and transmit diseases to farm animals and humans due to competition for food and breeding habitats (Sands and Wall 2017).

One reason for the astonishing biodiversity of dung inhabiting beetles is their temporal segregation both across the seasons (phenology), e.g., Kirk and Wallace (1990), Wassmer (1994) and over the aging and decomposition of dung (heterotrophic succession), e.g., Mohr (1943), Menéndez and Gutiérrez (1999). In this article, I will focus on heterotrophic succession, in which a resource like a freshly deposited dung pad is gradually consumed and decomposed by a series of species occurring at consecutive times during its decomposition (Nakamura 1975). Certain species occur early in this succession and some of them may stay only for a brief time, while others might stay longer, whereas a third group of species may colonize the resource at a later stage. For dung, a successional sequence of species was first described by Mohr (1943), who separated coprophilous species into 3 microseres. Since then, many studies in the Old and New World confirmed some of Mohr’s successional groups while others were reporting slightly to substantially different communities (Valiela 1974, Wingo et al. 1974, Koskela and Hanski 1977, Hanski 1980, Gittings and Giller 1998, Menéndez and Gutiérrez 1999, Richardson 2002, Sladecek et al. 2013, Nadeau et al. 2015, Rentz and Price 2016).

Despite the ecological and economic importance of coprophilous beetles, there are astonishingly few studies describing the succession of local dung beetle communities, especially in North America. Until now, no other research provided data on the successional segregation of dung beetles in Michigan and very few, mostly older studies provide successional data from any Midwestern state (Mohr 1943, Kessler and Balsbaugh 1972, Wingo et al. 1974). One objective of this study was, therefore, to add data for the coprophilous succession in the American Midwest. In addition to the much better-studied effects of the climate crisis on the seasonality of life processes including the phenology of communities (Renner and Zohner 2018, Damien and Tougeron 2019), there is emerging evidence showing that global warming also changes the succession of species after a disturbance or during heterotrophic processes (Dijkstra et al. 2010, Chang and Turner 2019). The second purpose of this study was, therefore, to provide currently missing baseline data describing the early stages of heterotrophic succession for the Midwest region enabling science to follow changes in the heterotrophic succession and decomposition of dung (Ott et al. 2012, Glassman et al. 2018) that could have substantial impact on soil fertility (Brevik 2013, Pugnaire et al. 2019) and the ability of soils to function as a carbon sink (Follett et al. 2012, Huang et al. 2019, Paustian et al. 2019). Almost all studies of the heterotrophic succession of dung so far collected dung pads (with the adjacent soil layer) after they were exposed for a certain amount of days. Such methods collect cumulative samples, which do not necessarily represent the attractiveness of a dung pad for colonization after the passed exposure time but provide information on how long the resource stays attractive (or tolerable) for already inhabiting beetles. In addition, the currently prevalent method is not able to compare the same individual dung pads between exposure times but rather assumes that different pads behave coherently. The third objective of this study was the collection of data from subtractive or exclusionary samples to deduce the relative attractiveness of dung after a 2-d versus 3-d exposure.

## Materials and Methods

### Location

**Fig. 1.**
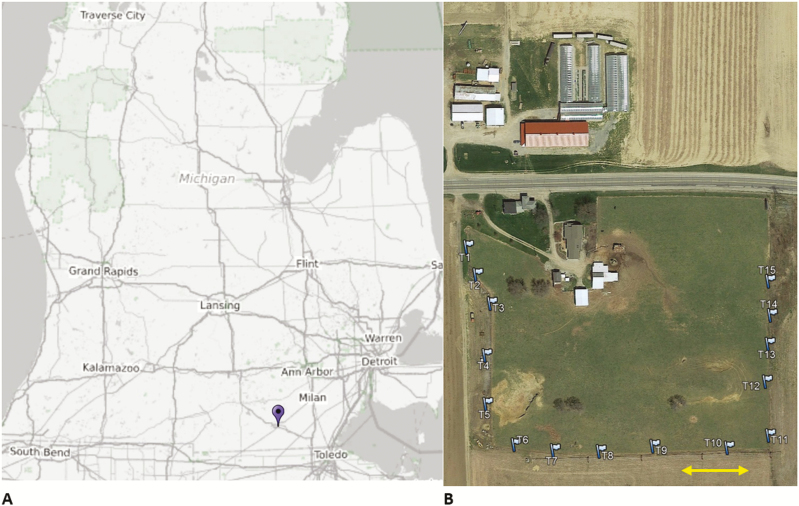
(A) Study location in Michigan (pin) and (B) trap array (flags) along the fence line of Carpenter Farm. The arrow indicates 50 meters.

**Fig. 2.**
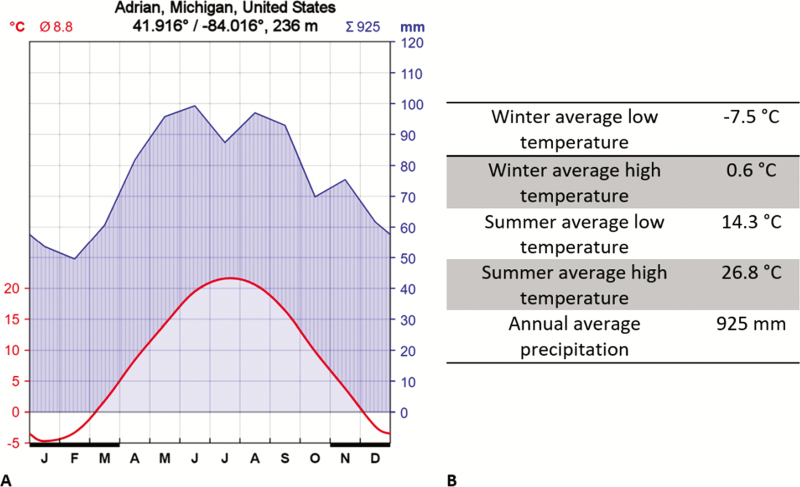
(A) Walter-Lieth climate diagram for Adrian, Michigan, based on monthly normal for ADRIAN 2 NNE, MI US, GHCND: USC00200032 from NOAA’s National Centers for Environmental Information (NCEI) plotted by the web application http://www.activevb.de/members/pjotrc/climograph/Climograph.html. The upper curve provides monthly average precipitation with the average yearly sum provided in the upper right of the diagram; the lower curve provides monthly average temperature with the yearly average temperature in the upper left. Dark bars above the initials of the months show regular frost months. (B): Long-term climate data for Adrian, Michigan based on the same data as in (A).

**Fig. 3.**
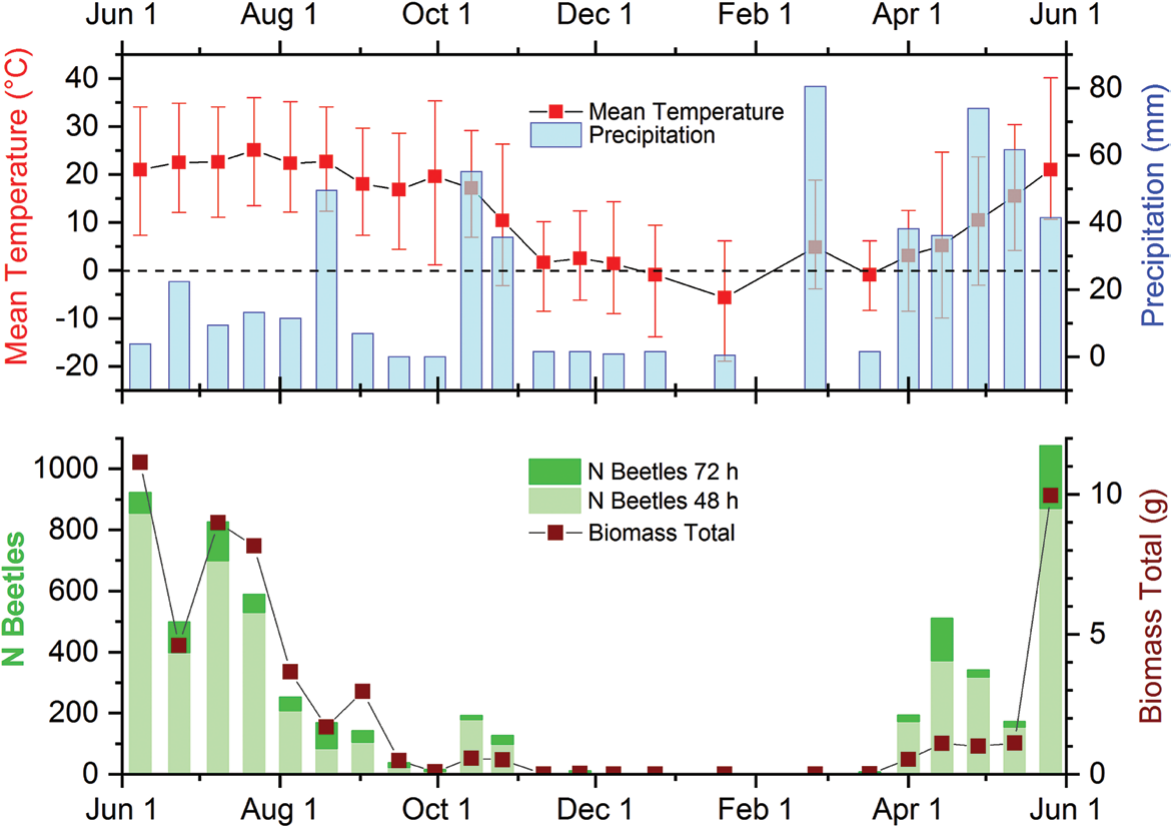
Weather data, beetle abundance, and biomass in the time course of the study. Upper panel: Mean (red squares) and high and low temperatures (red error bars) and precipitation (blue columns) during the sampling days. The dashed line indicates the freezing point. Lower panel: Beetle abundances as stacked columns with bright green indicating beetle abundances after 48-h exposure and dark green portions showing beetle numbers after 72 h of exposure. The wine-colored line and squares display beetle biomass collected on the sampling days.

I sampled coprophilous beetles from Carpenter Farm (41.874°, −84.010°) located approximately 2 miles southeast of Adrian, in Madison Township, Lenawee County, Michigan, United States. The pasture area is about 3.8 ha, surrounded by crop field on three sides and a major street on one side and elevated about 243 m above sea level. There are four small trees on the pasture and some farmhouses and barns close by (Fig. 1). The pasture area was grazed year-round for at least 25 yr. During my study, about 45 cows, heifers and calves of Black Angus and Hereford-Angus breeds roamed the 3.8 ha pasture. Since 2013, the cattle are moved to a larger more productive pasture in mid to late spring and is replaced by a small herd of goats, a pony and a donkey; cattle return to the farm in late autumn (Table 1). The soil types of Carpenter Farm are St. Clair loams (fine, illitic, mesic oxyaquic Hapludalfs) and Plainfield and Ottawa loamy sands (mixed, mesic typic Udipsamments) (SoilWeb-Earth 2020). Long-term climate data (Weatherbase 2020) for Adrian, MI (3 km NW of the sampling sites) identify average high and low temperatures in the coldest months (January and February) of 0 and −8°C, respectively. Average high and low temperatures in the hottest month (July) are 29 and 15°C, respectively. Average annual precipitation is 925 mm (Fig. 2). Weather conditions during the sampling days were recorded by the Weather Underground station KMIADRIA10 (Weather Underground 2020), which is located about 1.6 km south of the study area and is shown in Fig. 3. Forage species found on the pasture are mainly *Lolium spec.* (L.) (Poales: Poaceae), *Festuca arundinacea* (Schreb.) (Poales: Poeaceae), and native grasses. In winter, hay and pumpkins supplemented the diet. No growth promoters or antibiotics were used on a regular basis.

**Table 1.** Number and time intervals for the presence of grazing animals on Carpenter Farm

Date	Cows	Heifers	Calves	Other	Total
**Before 17 Apr. 2017**	**22**	**4**	**20**	**0**	**46**
**17 Apr. 2017**	**22**	**0**	**20**	**0**	**42**
**27 May 2017**	**4**	**0**	**3**	**0**	**7**
**4 June 2017**	**0**	**0**	**0**	**0**	**0**
12 June 2017	0	0	0	13 goats, 1 pony	14
1 Aug. 2017	0	0	0	18 goats, 1 pony	19
6 Oct. 2017	4			18 goats	22
4 Nov. 2017	26	0	0	0	26
8 Mar. to 3 Apr. 2018	26	0	24	0	50
28 Apr. 2018	6	0	6	0	12
8 May 2018	0	8	0	0	8
23 May 2018	0	0	0	0	0
25 May 2018	0	0	0	10 goats, 1 pony, 1 donkey	12

Bold rows are before the first sampling.

### Data Collection

To obtain coprophilous beetles, I setup 15 pitfall traps located just outside the fence line on three sides of the pasture (Fig. 1). Pitfall traps consisted of a 1-gallon (3.785 liters) high-density polyethylene bucket (opening diameter 0.203 m) buried into the ground allowing the rim of the bucket to stick out approximately 2 cm above the ground to exclude/reduce bycatches of epigean insects and to prevent surface water influx. Buckets were filled with approximately 750 ml water containing two tablespoons of sodium chloride and one tablespoon of liquid odorless soap. In winter, the saltwater and soap mix was replaced by a 1:1 mixture of water and a nontoxic high-performance antifreeze with a freezing point of −46°C. The buried buckets were covered by a 19 mm wire mesh to exclude/reduce bycatches of small mammals; a rain guard prevented/reduced water influx during precipitation. I baited the traps with approximately 400 g of fresh uninhabited cattle dung collected on site or a nearby farm and sampled twice per month between 8:00 a.m. and 5:00 p.m. depending on season and weather. Biweekly sampling started on 8 June 2017 and continued until 27 May 2018. I collected beetles from the bucket by straining the content through a No. 20 mesh (0.841 mm opening) after approximately 48 h without tampering with the bait, reset the traps with the bait exposed for 48 h and collected once again after 72 h. During one sample week, the second sample was taken after 96 h (25 December 2017). As the samples on two extreme winter days in December with temperatures below −20°C and a thick snow cover of more than 30 cm were uninhabited, I only collected one sample per month for January and February.

I preserved the samples in Scheerpeltz solution (70% ethanol, 5–10% acetic acid, 15–20% distilled water) and identified Scarabaeoidea using the keys in Freude et al. (1969), Lohse and Lucht (1992), Gordon and Skelley (2007), Skelley (2007a, b, c), Howden and Cartwright (1963), Ratcliffe (1991), Stebnicka and Lago (2005), and Wegner and Niemczyk (1979). For Hydrophilidae determination, I used Freude et al. (1971), Smetana (1978), Hansen (1987), Smetana (1988), and Lohse and Lucht (1989). Voucher specimens were placed in the insect collection of Siena Heights University, Department of Biology, Adrian, MI. To estimate the biomass of beetles, I used the average dry weight of 10 randomly selected individuals, or obtained values from Koskela and Hanski (1977), Lumaret and Kirk (1987), Wassmer (1991), and Šlachta et al. (2008). For a few species, I calculated biomass by using the dry weights of species with similar body length and shape (Table 2). I assigned species to be native or adventive according to the literature cited for beetle identification as well as Paulian (1959), Horion (1974), and Wallace (1962) for the Old World, and Bugguide (2013) for the New World.

**Table 2.** Numbers of dung beetles sampled from Carpenter Farm near Adrian, Michigan between June 2017 and May 2018

Species	Origin	Guild	Weight (mg)	*N* 48 h	*N* 96 h	Total	48 h (%)	72 h (%)	*P* (ChiSq)
Scarabaeidae									
Aphodiinae									
*Alloblackburneus lentus* (Horn)	n	D	0.8^*a*^	43	41	84	51	49	0.827
*Alloblackburneus rubeolus* (Palisot de Beauvois)	n	D	1.5^*a*^	31	12	43	72	28	0.004
*Aphodius fimetarius* (L.)	a	D	7.4^*a*^	12	13	25	48	52	0.841
*Ataenius spretulus* (Haldeman)	n	R	2.4^*a*^	1	3	4	25	75	0.317
*Blackburneus stercorosus* (Melsheimer)	n	D	1.1^*a*^	18	9	27	67	33	0.083
*Calamosternus granarius* (L.)	a	D	3.4^*c*^	29	5	34	85	15	0.000
*Chilothorax distinctus* Müller	a	R	2.8^*c*^	1,096	154	1,250	88	12	0.000
*Colobopterus erraticus* (L.)	a	R	9.1^*a*^	529	42	571	93	7	0.000
*Dialytes truncatus* (Melsheimer)	n	D?	4^*f*^	4	1	5	80	20	0.180
*Labarrus pseudolividus* (Balthasar)	n	D	2.8^*f*^	47	10	57	82	18	0.000
*Melinopterus prodromus* (Brahm)	a	R	4.6^*d*^	2	1	3	67	33	0.564
*Oscarinus rusicola* (Melsheimer)	n	D	2.1^*a*^	253	103	356	71	29	0.000
*Otophorus haemorrhoidalis* (L.)	a	D?	3.1^*a*^	74	24	98	76	24	0.000
*Pseudagolius bicolor* (Say)	n	D	5.1^*f*^	2	0	2	100	0	0.157
Melolonthinae									
*Maladera castanea* (Arrow)	a	NC?	10^*f*^	28	12	40	70	30	0.011
Rutelinae									
*Anomala innuba* (F.)	a	NC	10^*f*^	1	0	1	100	0	0.317
Scarabaeinae									
*Onthophagus hecate* (Panzer)	n	R	17.2^*a*^	307	57	364	84	16	0.000
*Onthophagus nuchicornis* (L.)	a	R	15.3^*a*^	711	44	755	94	6	0.000
*Onthophagus pennsylvanicus (*Harold)	n	R	3.8^*a*^	859	185	1,044	82	18	0.000
*Onthophagus taurus (*Schreber)	a	R	25.1^*a*^	336	36	372	90	10	0.000
*Onthophagus tuberculifrons* (Harold)	n	R	3.8^*f*^	17	4	21	81	19	0.005
*Phanaeus vindex* (MacLeay)	n	R	286.7^*a*^	36	0	36	100	0	0.000
Geotrupidae									
*Geotrupes opacus* (Haldeman)	n	R	163.4^*f*^	4	1	5	80	20	0.180
Ochodaeidae									
*Xenochodaeus americanus* (Westwood)	n	NC	10^*f*^	1	0	1	100	0	0.317
Hydrophilidae									
*Cercyon atricapillus* (Marsham)	a	D	0.3^*f*^	3	1	4	75	25	0.317
*Cercyon haemorrhoidalis* (F.)	a	D	0.9^*d*^	142	52	194	73	27	0.000
*Cercyon praetextatus* (Say)	n	D	0.9^*f*^	1	0	1	100	0	0.317
*Cercyon pygmaeus* (Illiger)	a	D	0.2^*b*^	20	13	33	61	39	0.223
*Cercyon quisquilius* (L.)	a	D	0.6^*b*^	177	111	288	61	39	0.000
*Cercyon terminatus* (Marsham)	a	D	0.3^*b*^	8	1	9	89	11	0.020
*Cryptopleurum americanum* (Horn)	n	D	0.3^*f*^	2	0	2	100	0	0.157
*Cryptopleurum crenatum* (Kugelann)	a	D	0.6^*b*^	3	9	12	25	75	0.083
*Cryptopleurum minutum* (F.)	a	D	0.4^*e*^	9	7	16	56	44	0.617
*Cryptopleurum subtile* (Sharp)	a	D	0.4^*f*^	10	6	16	63	38	0.317
*Sphaeridium bipustulatum (*F.)	a	D	5^*b*^	61	3	64	95	5	0.000
*Sphaeridium lunatum* (F.)	a	D	13.6^*b*^	82	6	88	93	7	0.000
*Sphaeridium scarabaeoides* (L.)	a	D	10.5^*b*^	142	2	144	99	1	0.000
Total				5,101	968	6,069			

Origin: n/a = native (n) or adventive (a) species for the Nearctic region; Weight: dry weight of beetles in mg; *N* = number of individuals collected; Species Totals: % = relative abundance of species in either exposure class as percentage of total number sampled from both exposures; *P* (ChiSq) indicates the significance of a χ ^*2*^ test comparing observed vs. expected frequencies for each species. Applying a Holm-Bonferroni Sequential Correction for multiple tests sets the significance level to *P* < 0.002. Guilds: D = dweller, R = relocator, NC = non-coprophilous. Dry weight of beetles in mg obtained by averaging dry weights of 10 specimen^*a*^ or estimated using values provided by Koskela and Hanski (1977)^*b*^, Lumaret and Kirk (1987)^*c*^, Wassmer (1991)^*d*^, Šlachta et al (2008)^*e*^ or calculated using species with similar body length and body shape^*e*^.

I classified all coprophilous beetles into two functional groups by larval and adult ecology:

1. Relocators – sensu Sladecek et al. (2013), whose larvae do not occupy the dung pad at any stage of their development; instead, they ‘relocate’ their larvae away from the dung pads. This includes paracoprid Scarabaeinae (Doube 1990) and Aphodiinae that oviposit in the soil away from the dung pad – in this study the frequently found species *Chilothorax distinctus* (Müller) (Coleoptera: Scarabaeidae) and *Colobopterus erraticus* (L.) (Coleoptera: Scarabaeidae) and the rare species *Melinopterus prodromus* (Brahm) (Coleoptera: Scarabaeidae) (Christensen and Dobson 1976, Rojewski 1983, Vitner 1995, Gittings and Giller 1997, Vitner 1998). In addition, the suspected turf pest *Calamosternus granarius* (L.) (Coleoptera: Scarabaeidae) might also be considered a relocator (Sears 1978, Smitley et al. 1998) although the species has also been reared from cattle dung (Gittings and Giller 1997, Floate 1998).
2. Dwellers used synonymous to endocoprids (Doube 1990), where the entire larval development or at least the last stages of it occur in the dung pad. In contrast to Sladecek et al. (2013), who included the endocoprid Hydrophilidae as a separate guild, I included them also as dwellers as I do not expect a major impact of predatory Hydrophilidae larvae within the short successional span in this study of just 3 d.

### Data Analyses

The study design provided two environmental parameters whose influence on the species community (SC) could be analyzed:

1. Phenology (seasonality) 26 biweekly samples from the pooled 15 pitfall traps or as 12 monthly samples with two biweekly samples pooled together. Analyses of these data will be published elsewhere.
2. Succession (exposure time) from the pooled 15 pitfall traps collected after 2 d exposure, when colonization can be expected to be at maximum (Koskela and Hanski 1977) and a pooled late sample collected after 3 d.

**Fig. 4.**
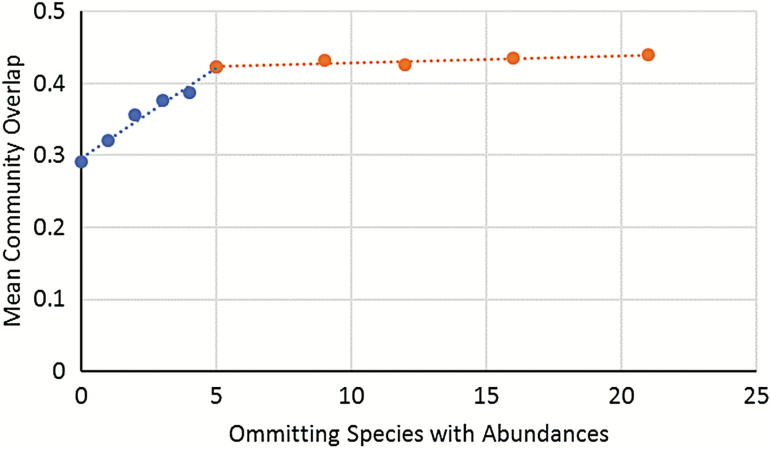
Graphical analysis of the influence of omitting rare species on the mean community overlap.

I visualized data using OriginLab Origin Pro 2019 and computed standard statistical tests to compare data against a χ ^2^ distribution and for comparisons of group means, SDs, and SEs (Siegel and Castellan 1988, Moore et al. 2013, Zar 2018). Unless specifically stated, means are presented together with their standard deviation (mean ± SD). Dominance structures of the SC were based on the dominance classes defined by Engelmann (1978): eudominant (ed) = 32–100%, dominant (d) = 10–31.9%, subdominant (sd) = 3.2–9.9%, recedent (r) = 1.0–3.19%, subrecedent (sr) = 0.32–0.99% and sporadic (s) = <0.32%. I decided on the Brillouin index as a measure of α-diversity and its associated evenness measure in analyses based on abundance and the Shannon-Wiener function and evenness in analyses based on biomass as suggested by Krebs (2014). I also calculated the true diversities as the exponential of the Shannon-Wiener entropy -exp (H_Shannon) and the effective number of species following Jost (2006). Similarities and dissimilarities between phenological and successional groups were calculated using unweighted paired-group cluster analyses (UPGMA) based on the percent similarity coefficient (Renkonen index, Renkonen 1938) or Pianka’s niche overlap index (Pianka 1973). To minimize a disproportional influence of rare species on the cluster analyses of niche overlap, I analyzed how omitting rare species one by one according to their abundances influenced mean community overlap (Fig. 4). As the slope of the linear regression decreased substantially at an abundance of 5, I decided to omit the 8 rarest species with abundances below 5. Diversities and overlaps were computed in PAST version 3.24 (Hammer et al. 2001), Community Ecology Parameter Calculator 1.0 (ComEcoPaC) (Drozd 2010), the Microsoft Excel add-in Diversity (Burn 2000), MVSP (Kovach 2010), and Ecological Methodology 7.3 (Krebs and Kenney 2016). The mean degree of community overlap was calculated using all possible pairs of species on the matrix of species and niche dimensions (succession and phenology, respectively). To assess the significance of the means, the degree matrices were randomized (*n* = 10,000) according to the algorithm RA3 (Winemiller and Pianka 1990) for discrete niche categories (exposure/succession) to create a null distribution of overlaps. A two-tailed significance was determined by comparing the randomized values with the respective observed distribution at α = 0.05. Significant overlap is indicated if the observed overlap is significantly greater than the randomizations, whereas segregation is indicated by significantly lower overlaps than that expected by chance (Albrecht and Gotelli 2001). The randomizations and the significance of the models R2, R3, and R4 were generated using the software EcoSim 7.71 (Gotelli and Emsinger 2005). The reliability of cluster analyses was confirmed by the cophenetic correlation coefficient, which indicates if a dendrogram preserved the pairwise distances between the examined samples sufficiently well (Sokal and Rohlf 1962).

## Results

### Species Composition

Over the entire year, I caught 6,069 specimens comprising 24 species of Scarabaeoidea and 13 species of Hydrophilidae (Table 2). The majority of the species of Scarabaeoidea are native to North America (14 out of 24), whereas nine species are introduced. In contrast to this, 11 of the 13 species of Hydrophilidae are introduced and only two species, *Cercyon praetextatus* (Say) (Coleoptera: Hydrophilidae) and *Cryptopleurum americanum* (Horn) (Coleoptera: Hydrophilidae) are considered native to North America. Table 2 also provides the ecological guilds into which the species can be included. Three species of Scarabaeoidea, the Melolonthinae *Maladera castanea* (Arrow) (Coleoptera: Scarabaeidae), the Rutelinae *Anomala innuba* (F.) (Coeleoptera: Scarabaeidae), and the Ochodaeidae *Xenochodaeus americanus* (Westwood) (Coeleoptera: Ochodaeidae) are not consistently associated with dung and are therefore attributed as non-coprophilous. I could not find distinctive information about the Aphodiinae *Dialytes truncatus* (Melsheimer) (Coleoptera: Scarabaeidae) and therefore list this species as a probable dweller (D?).

### Dominance Structure of the SC

**Fig. 5.**
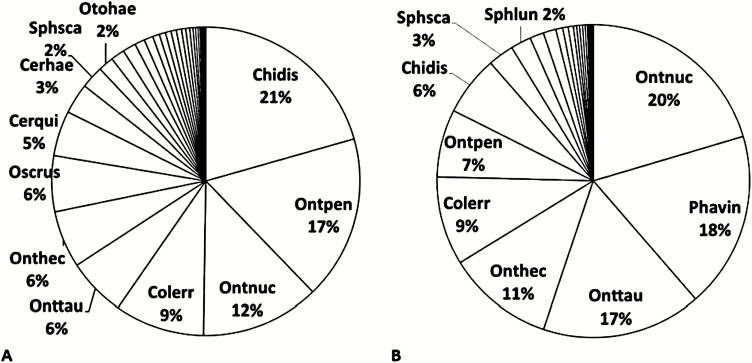
Dominance structures of the entire SC based on (A) beetle abundances and (B) beetle biomass. Note that 70% of the total beetle abundance were relocators (the 6 most common species), while 90% of the total beetle biomass came from relocators (the 7 most common species). Abbreviations: First three letters and the last three letters denote genus and species, respectively.

The SC was dominated by relocators that contributed more than 70% to the total beetle count and made up almost 90% of the total beetle biomass (Fig. 5). The largest relocator, *Phanaeus vindex* (MacLeay) (Coleoptera: Scarabaeidae), is more than 20 times larger than the largest dweller, the Hydrophilidae *Sphaeridium lunatum* (F.) (Coleoptera: Hydrophilidae), and more than 30 times heavier than the largest Aphodiinae, the also relocating *C. erraticus*. Despite its rarity of just 36 specimens, *P. vindex* was the second most dominant beetle in biomass.

### Succession/Exposure

The pooled samples collected after 48 h contained 5,101 beetles (84% of the grand total) and all 37 species, whereas the pooled samples collected after 72 h contained only 968 beetles (16% of the grand total) and 31 of the 37 species. In the current study, most species that were abundant enough to allow statistical testing were found in significantly higher numbers in the 48-h sample than in the 72-h sample (χ ^2^ tests, *P* < 0.05—applying a Holm-Bonferroni Sequential Correction for multiple tests reduces the significance threshold to *P* < 0.002, Table 2). Only *Alloblackburneus lentus* (Horn) (Coleoptera: Scarabaeidae) and *Aphodius fimetarius* (L.) (Coleoptera: Scarabaeidae) occurred approximately in equal numbers in 48 and 72 h exposed dung (43 vs. 41 and 12 vs. 13). All other species that were approximately evenly distributed over the 48-h and 72-h exposure classes were too rare for conclusive statistics. No species was found significantly more often in 72 h exposed dung (Table 2). Biodiversity was very similar between the pooled samples collected after 48 h and 72 h and showed about 71% similarity (Renkonen-Index 0.71, Table 3).

**Table 3.**
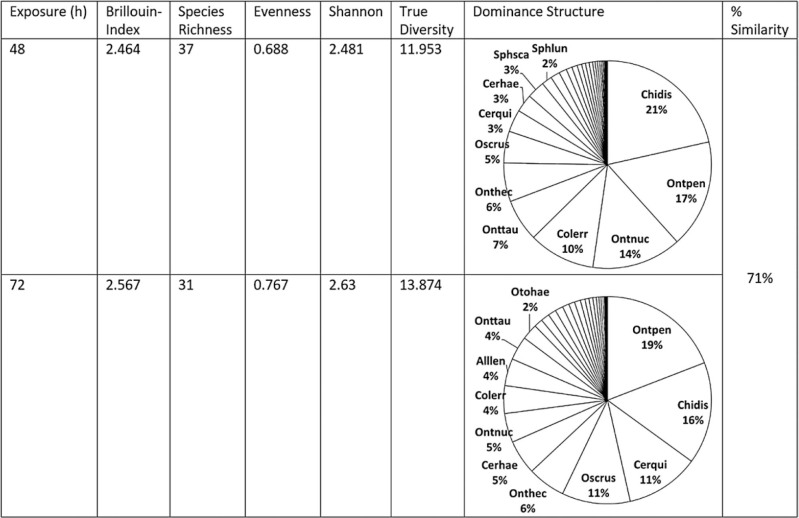
Biodiversity and similarity metrics for the beetle communities in cow dung exposed for 48 h and 72 h. True diversity was calculated following Jost (2006), % similarity was determined as the Renkonen index (Krebs 2014).

**Fig. 6.**
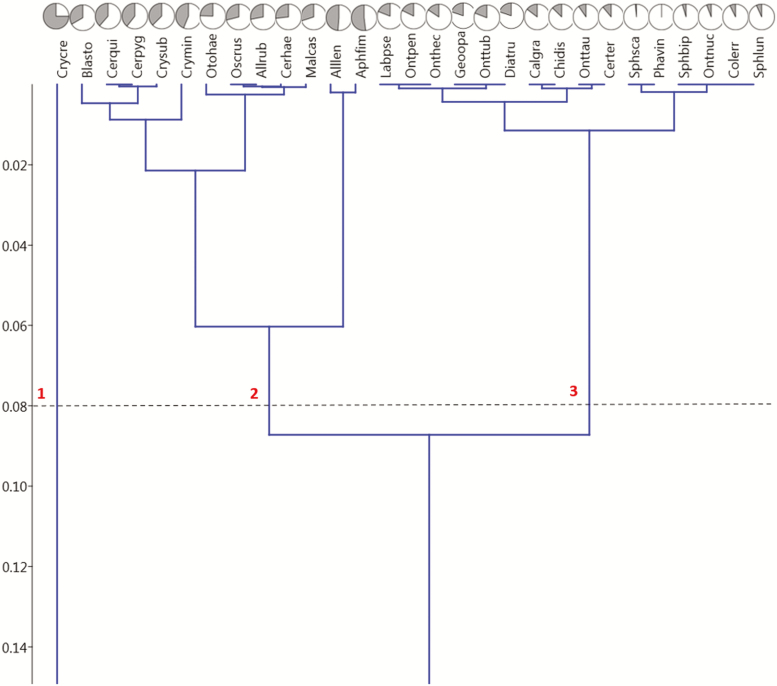
Unweighted Paired-group (UPGMA) cluster analysis of the successional species overlap based on Pianka’s index. The *y*-axis displays distance from 100% overlap, e.g., 0.02 translates to 98% overlap. Only species that were collected at least 5 times were included. Species abbreviations start with the first 3 letters of the genus followed by the first 3 letters of the specific epithet. Refer to Table 2 for a list of all species in this study. For each species, the pie charts on top of their names show the proportions of beetles found after 48 h (white sectors) and 72 h (grey sectors). At an overlap of 92%, 3 clusters are distinguishable. The cophenetic correlation coefficient was 0.8371 showing that the dendrogram preserved the pairwise distances between the examined samples sufficiently well indicating highly reliable results of the cluster analysis.

The UPGMA cluster analysis of successional species similarities based on Pianka’s niche overlap index confirmed a high degree of similarity with three distinct clusters forming at 92% similarity (Fig. 6). *Cryptopleurum crenatum* (Kugelann) (Coleoptera: Hydrophilidae) appeared alone as this species was the only one that occurred more frequently in samples collected after 72 h (9 vs. 3 after 48 h), although this difference was not significant (*Χ*^2^ (1, *n* = 12) = 2.08, *P* < 0.149). The third cluster included species that were found almost exclusively in 48 h exposed samples (more than 80%, mean 88.51 ± 6.43%), whereas cluster 2 contained species that were found in 72 h at least in 25% of the samples up to even quantities in both exposure classes (mean 64.46 ± 8.94%). The proportion of beetles in the early sample (48 h) was significantly higher in cluster 3 compared to cluster 2 (*t*(25) = 2.1, *P* < 0.00001). The weight distribution of species in cluster 2 (median 0.85 mg) was significantly lower than in cluster 3 (median 7.05 mg) (Mann–Whitney *U* = 9, *n*_1_ = 12, *n*_2_ = 15, *P* < 0.05 two-tailed). The analysis of niche overlap based on null models suggested a higher mean overlap (observed mean = 0.92233) than what was expected by chance in model RA3 (Winemiller and Pianka 1990); the mean of simulated indices was 0.74331 ± 0.00016 (*P* (observed ≥ expected) = 0.00000) suggesting significant overlap of the community. The reliability of the cluster analysis was confirmed by a cophenetic correlation coefficient of 0.8371.

Grouping species together into taxonomic and functional groups (guilds) revealed that relocators showed an almost three times stronger avoidance of aged dung (72 h) compared to dwellers (12 vs. 30%, Table 4). This difference was significant (one-way ANOVA *F*(1,26) = 7.15, *P* = 0.013). Hydrophilidae did not differ significantly from Aphodiinae dwellers in their tolerance of older dung (29 vs. 30%); however, the three species of the genus *Sphaeridium* showed a strong preference for fresh dung (only 4% found in the 72-h sample) compared to 29% for the genus *Cercyon* and 52% in *Cryptopleurum* (Table 4, one-way ANOVA *F*(2,7) = 8.91, *P* = 0.012). The difference between *Cryptopleurum* and *Sphaeridium* was significant (Tukey test, *P* < 0.05).

**Table 4.** Percentage of beetles caught in the late successional sample (72 h) in various taxonomic and functional groups

Grouping	N	Mean	SE Mean	StDev	Minimum	Q1	Median	Q3	Maximum
Aphodiinae	11	26.13	4.3	14.26	7.36	14.71	24.49	33.33	52
Dwellers	9	29.75	4.37	13.1	14.71	18.77	27.91	41.07	52
Relocators	2	9.84	2.48	3.51	7.36	*	9.84	*	12.32
Scarabaeinae	6	11.32	3.06	7.49	0	4.37	12.67	18.05	19.05
Geoptrupidae	1	20	*	*	20	*	20	*	20
Hydrophilidae	10	28.5	7.28	23.03	1.39	6.29	32.15	40.48	75
Cercyon	4	28.96	6.61	13.21	11.11	15.03	32.67	39.18	39.39
Cryptopleurum	3	52.1	11.6	20.1	37.5	37.5	43.8	75	75
Sphaeridium	3	4.3	1.58	2.74	1.39	1.39	4.69	6.82	6.82
All dwellers	19	29.09	4.24	18.49	1.39	14.71	27.91	39.39	75
All relocators	9	11.96	2.26	6.79	0	6.59	12.32	18.38	20

*N*, number of beetle species in the taxonomic or functional group; SE, standard error of mean; StDev, Standard deviation; Q1, Q3, First and third quartile.

*Sample size is too small to calculate statistics.

## Discussion

### Beetle Abundances and Species Composition

The present study is based on 6,069 specimens comprising 24 species of Scarabaeoidea and 13 species of Hydrophilidae and was sampled biweekly over an entire year, including the winter months. The only other study on coprophilous beetles in the immediate area was conducted by myself between March 2012 and June 2013 on two different farms approximately 10 miles west of the current study site (Wassmer 2014). That study resulted in a much lower total beetle count of 1,770 beetles representing 17 species of Scarabaeidae and nine species of Hydrophilidae. The lower total beetle count can be attributed to the different sampling method used—handpicking from dung found on the pastures with a pair of feather tweezers versus pitfall traps in this study. Nevertheless, the lower species richness of 7 Scarabaeoidea and 4 Hydrophilidae in my previous study surprises me as I consciously sampled all available microclimates (exposed and shaded areas) and two different types of dung—steer pads and sheep lumps. However, due to the relatively low sample size, it is possible that the sample did not include all species present in the area. The only other study from the Great Lakes region that I am aware of was conducted by Rounds and Floate (2012) in Lake City, Michigan, which is approximately 280 km NNW of Adrian (250 km further north). The authors collected 10,041 beetles in 20 taxa of Scarabaeoidea and Hydrophilidae using 40 biweekly sampled pitfall traps between May and October. Their higher total beetle numbers are expected due to using more than double the number of traps. It should have been even higher as a much larger herd of cattle was pastured at their site, and the cattle were not moved away to another pasture over the summer months, which was the case in the present study. One possible explanation could be the much smaller bait and trap size of only 75 g bait (as to approximately 400 g in the current study), and their use of cups of only 550 ml (as to 3.785-liter buckets).

The current study included all species found in my previous study from the Adrian, Michigan area besides the large Aphodiinae *Teuchestes fossor* (L.) (Coleoptera: Scarabaeidae), which was only found once in the previous study and 23 times (0.23%) in the N-Michigan study by Rounds and Floate (2012). The current study lacked the Geotrupidae *Geotrupes egeriei* (Germar) (Coleoptera: Geotrupidae) and *Geotrupes semiopacus* (Jekel) (Coleoptera: Geotrupidae). These species and the missing Aphodiinae *Agoliinus leopardis* (Horn) (Coleoptera: Scarabaeidae) and *Agoliinus manitobensis* (Brown) (Coleoptera: Scarabaeidae) are considered forest species. Forest patches occurred on the N-Michigan study site but not anywhere around Carpenter Farms. The only other species not detected in the present study were the Scarabaeinae *Copris fricator* (F.) (Coleoptera: Scarabaeidae) and *Melanocanthon nigricornis* (Say) (Coleoptera: Scarabaeidae), which should both occur in the area (Ratcliffe 1991, Nemes and Price 2015). On the other hand, the two regional reference studies by Wassmer (2014) and Rounds and Floate (2012) were missing several species of the current study, notably *P. vindex* and *Onthophagus tuberculifrons* (Harold) (Coleoptera: Scarabaeidae) and *Geotrupes opacus* (Haldeman) (Coleoptera: Geotrupidae). I assume a combination of climatic and soil factors, but according to the literature, these species could occur on the other two sites as well (Ratcliffe 1991, Nemes and Price 2015). In the current study, the black turfgrass Ataenius, *Ataenius spretulus* (Harold) (Coleoptera: Scarabaeidae) was found in much lower numbers than in my previous study (Wassmer 2014), was missing from Rounds and Floate (2012) and was found regularly in South Dakota (Kessler and Balsbaugh 1972). In my previous study based on handpicking, this species was often found at the interface between dung, vegetation, and soil (Wassmer 2014), which could explain why it was better represented by hand collection or whole dung pad collection, which commonly also includes the first few centimeters of soil under a dung pad.

Fourteen of 24 species of Scarabaeoidae and 2 of 13 species of Hydrophilidae are native to the New World. In comparing the species list of the current study to Kessler and Balsbaugh (1972) from South Dakota, the lower abundances or lack of many introduced and invasive species, especially of the *Onthophagus* (Latreille) (Coleoptera: Scarabaeidae) species *O. nuchicornis* (L.) (Coleoptera: Scarabaeidae) and *O. taurus* (Schreber) (Coleoptera: Scarabaeidae), and the Aphodiinae *C. erraticus* may indicate an earlier stage of invasions at the more northern location, the time difference of almost 50 yr between the records and the since then accelerating effects of the man-made climate crisis, especially in arctic and cold-temperate biomes (Roots 1989, Peng et al. 2019). Since the beginning of the millennium, *O. nuchicornis* was found in North Dakota (Tinerella and Fauske 1999), *O. taurus* was first found in Michigan in 2012 (Rounds and Floate 2012) and since about the same time, *C. erraticus* is known to occur as far north and west as Southern Alberta (Floate and Kadiri 2013, Floate et al. 2017). This highlights the importance of studies documenting species communities to reconstruct invasions and the effects of the climate crisis. In contrast to this, the Aphodiinae *C. distinctus* was described for the northern plains (Southern Alberta) as early as the 1920s (Seamans 1934). Kessler and Balsbaugh (1972) most probably missed this species in South Dakota due to collecting beetles outside the phenological position of this species at this latitude (Wassmer 2014).

### Dominance Structure of the SC

The SC was dominated by relocators that contributed more than 70% to the total beetle count and made up almost 90% of the total beetle biomass (Fig. 5). As it took seven species to reach 75% of the SC based on beetle abundances, and five species to reach 75% of the total biomass, the SC of Carpenter Farms is more evenly distributed than many European SCs, where often just three to four species make up 80% of the SC (reviewed in Sullivan et al. (2017b).

Several recent studies from many different parts of North America indicate that the number and dominance of native coprophilous Scarabaeoidea species increases in forests or other native vegetation (Simons et al. 2018, Bezanson 2019, Conover et al. 2019) and decreases with an increase in uniformity in man-made pastureland, especially in northern cold-temperate pastures such as S-Alberta, where the two most abundant species are the invasive non-native species *C. distinctus* and *O. nuchicornis*. If this correlation holds in a more systematic analysis, it would highly suggest conservation strategies following the land ‘sharing’ approach to increase biodiversity on agricultural land (Kremen 2015).

### Succession/Exposure

The sampling method I used for the early stages of succession was very different from almost all published studies so far. As I used the same untampered bait in my pitfall traps and just sampled the buckets of the pitfall traps below, I excluded beetles that arrived within 48 h at the dung pad from the 72-h sample. To my knowledge, the same method was only used in two other studies so far (Rentz and Price 2016, Simons et al. 2018). With this method, I cannot deduce if some of the beetles trapped within 48 h would have stayed in the dung pad until my 72-h sample or beyond. Most of the successional studies that I reference below collected the entire dung pads (with the underlying soil layer) after they were exposed for the specified time (usually between 1 and 30 d). Such samples are thus cumulative, while the method used in this study can be called exclusionary or subtractive. Cumulative samples do not necessarily represent the attractiveness of a dung pad for colonization after the passed exposure time but provide information on how long the resource stays attractive (or tolerable) for the already inhabiting beetles. In addition, the cumulative method is not able to compare the same individual dung pads between the exposure times but rather assumes that different pads behave coherently. In contrast to this, the method used in this study detects how attractive dung pads at the given exposure time were for new colonization but cannot address how long a resource stays attractive or tolerable to continued colonization. Despite these methodic differences, most species in this study followed patterns similar to those described by cumulative sampling methods. It is possible that removing beetles after 48 h depleted the pool of available beetles for recolonization in the 72-h sample. However, the same issue applies for all sampling methods that remove beetles from pastures, including traditional cumulative methods. I did some searching on this topic but could not find any literature about it.

### Abundance and Species Richness in the Exposure Classes

In the current study, the pooled 48-h samples contained 84% of the grand total (5,101 beetles), whereas the pooled 72-h samples contained only 16% of the grand total (968 beetles). A rapid decrease in the total abundance of inhabiting beetles over succession was found in almost all studies. Similar to the current study, Kessler and Balsbaugh (1972) collected only 28% of the total abundance of beetles after 72 h. The same trend was also reported by Sullivan et al. (2017b) for a site at Turkey’s Black Sea coast, where 78–97% of the total beetle abundances were found in dung pads exposed for 48 h followed by a sharp reduction of abundance in dung pads exposed for 96 h (their late sample). It is possible that deposited dung pads lose their attractiveness quickly, probably due to desiccation and nutrient decomposition (Sowig and Wassmer 1994, Frank et al. 2017). In contrast to the rapid decline in total beetle abundance, species richness in the current study diminished only from 37 to 31 species and the diversity of the pooled 72-h sample was slightly higher and more even than in the pooled 48-h sample. Both communities showed about 71% similarity (Renkonen-Index 0.71, Table 3). This corresponds well to Kessler and Balsbaugh (1972), who still found 22 of 23 species after 72-h but contradicts the results of Sullivan et al. (2017b), in which species richness followed the same trend as total beetle abundance and also plummeted from 17 species in their 48-h sample to 1–5 species in their 96-h sample. Dung pads seem to lose their attractiveness quickly, thus reducing total abundance from 48 h to 72 h substantially, but for most species, this seems to be a gradual process only excluding few species from the 72-h sample. It is known that low soil nutrient content facilitates the coexistence of more plant species (Tokeshi 1993, Zemunik et al. 2016) as suboptimal habitats tend to limit highly competitive species that would exclude inferior competitors. It is reasonable to apply this principle to decaying dung pads. This would explain why the 72-h sample could facilitate a slightly more even and more diverse SC. This assumption also agrees well to an application of the medium disturbance theory (Grime 1974, Connell 1978) to heterotrophic succession predicting a slightly increased diversity in the early mid-successional stages of heterotrophic succession (e.g., Sabu et al. 2006).

### Successional Species Differences

In the current study, most species were found in significantly higher numbers in the 48-h sample compared to the 72-h sample (χ ^2^ tests, *P* < 0.05, Table 2). Only *A. lentus* and *A. fimetarius* occurred approximately in equal numbers in 48- and 72-h exposed dung (Fig. 6, Table 2). No species was found significantly more often in 72-h exposed dung (Table 2). This result shows that for almost all species, dung pads lose their attractiveness for new colonization after just 2 d. Mohr (1943) listed *A. fimetarius* as a late successional species and part of his third microsere in Illinois, which was not contradicted in any later study I am aware of. The current study also suggests that *A. lentus* is a late successional species., probably equivalent to *A. fimetarius* as the two species were grouped together within cluster 2 and were distinctly apart from the other species of the cluster (see below).

### Cluster Analysis

A paired-group (UPGMA) cluster analysis of successional species similarities based on Pianka’s niche overlap index, confirmed a high degree of similarity between the species (Fig. 6). The high overlap in the SC reflects the fact that almost all species occurred in much higher numbers in fresh dung pads but did not completely avoid dung pads older than 48 h (Tables 2 and 3). However, the cluster analysis was sensitive enough to distinguish three clusters at 92% similarity.

### Clusters 1 and 2

The small Hydrophilidae *Cryptopleurum crenatum* stood isolated forming cluster 1 with only 43% similarity to any other species, as this species was the only one that occurred more frequently in samples collected after 72 h (9 vs. 3 after 48 h). However, this difference was not significant (*Χ*^2^ (1, *n* = 12) = 2.08, *P* < 0.149). Unfortunately, this result cannot be compared to other studies as no other successional study so far included *C. crenatum*. The SC included three other *Cryptopleurum* species, *C. minutum* (F.) (Coleoptera: Scarabaeidae), *C. subtile* (Sharp) (Coleoptera: Scarabaeidae), and *C. americanum*. The latter species, which is the only native Nearctic species, was found only twice and was therefore omitted from the cluster analysis (Fig. 6). The other two species were part of cluster 2 with 44% of *C. minutum* and 38% of *C. subtile* in 72-h exposed dung. Given the methodological differences between the current and most other studies described above, these values correspond well to Kessler and Balsbaugh (1972), who found 27% of *C. minutum* after 72 h in South Dakota, United States, Lee and Wall (2006), who found a successional mean occurrence (SMO, following the method of Hanski 1980) of 9.9 d in England, and Sladecek et al. (2013), who mention *Cryptopleurum* species to be part of the later phase of succession in the Czech Republic. As my successional sampling only lasted for 3 d and was discontinuous, I cannot closely compare my results to any of these findings. However, the fact that I found 56 and 62% of these late-successional species, respectively, after only 48 h of exposure indicates that the reported successional position for *Cryptopleurum* species is not caused by an increasing attractiveness of dung over the first 10 d but rather a prolonged stay in previously colonized dung pads.

Cluster two also included all but one species of *Cercyon*, the other genus of small coprophilous Hydrophilidae in this study, including *C. haemmorhoidalis* (F.) (Coleoptera: Hydrophilidae), *C. quisquilius* (L.) (Coleoptera: Hydrophilidae), and *C. pygmaeus* (Illiger) (Coleoptera: Hydrophilidae) occurring at percentages of 27, 39, and 39% in 72-h dung, respectively (Table 2, Fig. 6). Only *C. terminatus* (Marsham) (Coleoptera: Hydrophilidae) showed a stronger preference for fresh dung (89%) and was included in cluster 3 (below). Mohr (1943) included small Hydrophilidae dwellers of the genus *Cercyon*—*C. quisquilius*, *C. haemorrhoidalis*, and *C. pygmaeus* into his second microsere in Illinois, which corresponds well to the higher percentages I found these species in the more aged dung, and agrees also well to Kessler and Balsbaugh (1972), who found *Cercyon* species co-occurring together with *Cryptopleurum minutum* at around 25% in South Dakota. In their study from England, Lee and Wall (2006) included *C. haemorrhoidalis* and *C. quisquilus* into their group I (SMO 5.1 and 6.8 d, respectively) and separated them from *C. terminatus*, *C. pygmaeus*, and *Cryptopleurum minutum*, which were included in their group II with SMO’s of 8.2, 9.0, and 9.9 d, respectively. This agrees well to the difference that I saw between *C. haemorrhoidalis* and the other *Cercyon* species but is contrary to my results for *C. terminatus*.

In addition to small Hydrophilidae, cluster 2 also contained the Aphodiinae *Blackburneus stercorosus* (Melsheimer) (Coleoptera: Scarabaeidae), *Oscarinus rusicola* (Melsheimer) (Coleoptera: Scarabaeidae), *Alloblackburneus rubeolus* (Palisot de Beauvois) (Coleoptera: Scarabaeidae), and *Otophorus haemorrhoidalis* (L.) (Coleoptera: Scarabaeidae), which occurred in 72-h exposed dung at 33, 29, 28, and 24%, respectively. Finally, cluster 2 also included the more isolated species *Alloblackburneus lentus* and *Aphodius fimetarius*, which occurred in the older dung at 49 and 52%, respectively (Table 2, Fig. 6). For their site in South Dakota, Kessler and Balsbaugh (1972) reported *B. stercorosus* as one of the latest Aphodiinae to occur with 46% collected from cow pads after 48 h. In the current study, only 33% of the species was found in the late sample, which is more in the mid-range for Aphodiinae. Mohr (1943) listed *A. fimetarius*, *O. haemorrhoidalis*, and *O. rusicola* as part of the third microsere in Illinois. Whereas my results agree with *A. fimetarius* (52% in the 72-h sample), I found only 24% of *O. haemorrhoidalis* and 29% of *O. rusicola*, respectively, in the 72-h sample. It is likely that the higher percentages in the reference studies are the result of the cumulative nature of almost all other studies. In their study of montane pastures in N-Spain, Menéndez and Gutiérrez (1999) found that *O. haemorrhoidalis* had a SMO of 8.9 d in May, and 5.6 d in July. Despite showing less of a preference for aged dung, I found *O. haemorrhoidalis* to show the same trend towards older samples in May and early June and towards fresher samples in late June and July (Wassmer, unpublished results). Climatic factors might play an important role in this shift in both locations.

### Cluster 3

The third and largest cluster included species that were found almost exclusively (more than 80%) in 48-h exposed samples, notably all three *Sphaeridium* species and *Cercyon terminatus*, all Scarabaeinae of the genera *Onthophagus* and *Phanaeus*, and few Aphodiinae species that are mostly known to be relocators, a life strategy that is far less common for this subfamily of dwellers (Doube 1990, Sladecek et al. 2013). The early successional position found for these species in my current study corresponds generally well to previous research (Mohr 1943; Kessler and Balsbaugh 1972; Gittings and Giller 1998; Lee and Wall 2006; Sladecek et al. 2013; Sullivan et al. 2017a, b). This included the large Hydrophilidae dweller *Sphaeridium scarabaeoides* (L.) (Coleoptera: Hydrophilidae), for which 142 out of 144 specimens were found in fresh dung (99%). The other two *Sphaeridium* species in this study, *S. lunatum* (F.) (Coleoptera: Hydrophilidae) and *S. bipustulatum* (F.) (Coleoptera: Hydrophilidae), showed almost the same preference for freshly exposed dung with 93 and 95%, respectively (Table 2, Fig. 6). In contrast to this, Mohr (1943) listed the Hydrophilidae dweller *S. bipustulatum* as part of his second microsere in Illinois, United States but mentioned that the species was too rare to be conclusive. In England, Lee and Wall (2006) included all three species into their successional group I, but found the SMO to be 2.7 d for *S. scarabaeoides*, 5.0 d for *S. lunatum* and 5.3 d for *S. bipustulatum*. As my sampling only lasted for 3 d and was discontinuous, I cannot closely compare my results to these findings. However, the fact that all three species showed a diminished presence after 48 h in the current study indicates that the reported differences between the *Sphaeridium* species in cumulative samples are not due to an increasing attractiveness of dung over the first 5 d but rather a longer stay of *S. lunatum* and *S. bipustulatum* compared to *S. scarabeoides*. Otronen and Hanski (1983), found *S. lunatum* to peak consistently about 1 d later than *S. scarabaeoides*. The authors considered this small but consistent difference to be enough to have ecological consequences facilitating the coexistence of two otherwise very similar species. Interestingly, Hanski (1980) compared the differences between *Sphaeridium* species from two sites in the United States and two sites in Europe and concluded that the *Sphaeridium* species in the United States were successionally closer to each other than in Europe, which would explain the coincidence with my findings. Only *Phanaeus vindex* showed an even more pronounced preference for fresh dung with all 36 specimens retrieved from fresh dung pads (48 h). This species’ successional preference was only described in one other successional study from Maryland (Simons et al. 2018), in which the species was found in younger and older human dung (day 1 to day 5 with 1 of 15 beetles occurring as late as day 21). Interestingly, this study is one of only two other studies that used pitfall traps and did not replace the bait—like in the present study making the results highly comparable. The difference in the successional position of *Phanaeus vindex* might be due to differences in dung type (cattle vs. human) and/or habitat differences (pasture in the current study—forests in Simons et al. (2018). Forested habitats provide more shade keeping dung moist and potentially more attractive for longer periods of time. In addition, dung could also be scarcer in forests, increasing the tolerance for suboptimal resources (Wassmer 1995).

Other species from Mohr’s first microsere (Mohr 1943) found in the current study were the Aphodiinae *Chilothorax distinctus* and *Pseudagolius bicolor* (Say) (Coleoptera: Scarabaeidae), which was only found twice (both in 48-h exposed dung) and is thus not further discussed. *Chilothorax distinctus* was one of the most abundant species and 88% occurred in dung exposed for 48 h, thus corresponding well to Mohr’s findings. As the species does not complete its larval development in dung but rather in soil, it is considered a relocator (Vitner 1995). In their study on the succession of coprophagous dung beetles in various dung types in Ireland, Gittings and Giller (1998) confirmed *Colobopterus erraticus*, a palaearctic species that becomes increasingly abundant in North America as an early successional species. In the current study, 93% of the specimens were found in dung exposed for 48 h. This makes *C. erraticus* by far the Aphodiinae species with the strongest preference for freshly deposited dung. Interestingly, like *C. distinctus*, *C. erraticus* is not a dweller but a relocator (Rojewski 1983, Vitner 1998). Eighty five percent of the specimens of *Calamosternus granarius* were found in fresh dung and was, therefore, part of cluster 3 in the current study. In contrast to this, Gittings and Giller (1998) found *C. granarius* to be a mid-successional species. To my knowledge, the reproductive behavior of *C. granarius* is unknown and the species is therefore reported as a dweller, the default for Aphodiinae. However, the species is suspected to be a turf pest (Sears 1978, Smitley et al. 1998) and might well be a facultative relocator as it has been reared from cattle dung (Floate 1998). *Labarrus pseudolividus* (Balthasar) (Coleoptera: Scarabaeidae) was also a cluster 3 species with 82% in 48-h samples. Up to now, the successional position of this species was only described by Rentz and Price (2016), who also found this species concentrated after 1 d of exposure and fading out at 72 h to reach zero after 5 d. This species is reported to be a dweller depositing its eggs into dung pads and thus competing with fly larvae (Bertone et al. 2004). However, no specific evidence to support this statement was provided. As this species would be the only Aphodiinae dweller with a strong preference for fresh dung in cluster 3, it seems possible that this species could be a (facultative) relocator. This assumption is somewhat supported by Fiene et al. (2011). The last Aphodiinae in cluster 3 was *Dialytes truncatus*, from which four of the five specimens were found in fresh dung pads (80%). Little is known about this suspected deer and forest/savanna species (Gordon and Skelley 2007), including its reproductive life strategies. A successional separation of Aphodiinae into early relocators and late dwellers was previously suggested by Menéndez and Gutiérrez (1999).

Kessler and Balsbaugh (1972) found 77 and 86% of the two native *Onthophagus* species *O. hecate* (Panzer) (Coleoptera: Scarabaeidae) and *O. pennsylvanicus* (Harold) (Coleoptera: Scarabaeidae), respectively, in 48-h exposed dung. This corresponds well to 84 and 82% in the current study. No study until now reported the successional position of *O. tuberculifrons* (Harold) (Coleoptera: Scarabaeidae), which was found 81% in fresh dung. The introduced *Onthophagus* species *O. nuchicornis* and *O. taurus* showed an even stronger preference for fresh dung with 94 and 90% of specimens found in the 48-h exposure group. This corresponds very well to Sullivan et al. (2017b), who did not find any *Onthophagus* species in dung exposed longer than 48 h in their study at the Turkish Black Sea coast. *Onthophagus* species are paracoprid and thus also relocators. Four of five specimens of *Geotrupes opacus* (Haldeman) (Coleoptera: Scarabaeidae), the only Geotrupidae in this study, occurred in fresh dung and warranted the inclusion of this species in cluster 3. Geotrupidae are generally considered to be paracorid relocators.

### Comparison Between Clusters 2 and 3: Dwellers Versus Relocators

The relocators in the third cluster did not only show a significantly higher attraction to fresh dung (mean 88.51 ± 6.43%) compared to cluster 2 (mean 64.46 ± 8.94%, two-tailed *t*(25) = 2.1, *P* < 0.00001) but relocators (median 7.05 mg) were also significantly larger than dwellers (median 0.85 mg, Mann–Whitney *U* = 9, *n*_1_ = 12, *n*_2_ = 15, *P* < 0.05 two-tailed). It is possible that the almost exclusive preference of relocators for freshly deposited dung is caused by the need to relocate dung to their eggs in tunnels or shallow galleries in the soil or between grassroots before the resource gets too dry and before substantial competition from other coprophagous beetles and Diptera occurs. It is possible that the larger average size of relocators allows them to relocate dung more effectively but could also be a reason why they cannot be dwellers as they would face more competition than smaller species and might be more sensitive to changing abiotic factors during the aging of dung. However, this could not explain why large Hydrophilidae dwellers of the genus *Sphaeridium* have a pronounced preference for fresh dung placing them in cluster 3 dominated by relocators whereas most small Hydrophilidae of the genera *Cercyon* and *Crytopleurum* were late-successional species placed in cluster 2 together with other dwellers. *Sphaeridium* adults occurs almost instantly after dung pads were deposited and lay eggs rapidly, which develop into larvae within 2–3 d. The fast colonization and development are believed to be an adaptation to the rapid life cycle of their dipteran prey species that develop from eggs to adults in about 5 d (Otronen and Hanski 1983, Sowig et al. 1997, Archangelsky 1999). It is possible that the likewise predatory larvae of most small Hydrophilidae do not dependent on rapidly developing prey species and therefore do not need to arrive early at a dung pad. In addition, their smaller size might allow them to live and feed longer in aging dung pads compared to the 20 times larger *Sphaeridium* species.

The results of the cluster analysis were confirmed when grouping species together into taxonomic and functional groups (guilds).

The lack of finding a qualitative differentiation into microseres (Mohr 1943) could be linked to the shortness of the sampling protocol of just 3 d, which might not allow for microsere differentiation. In addition, my subtractive sampling protocol excluded most of the specimens and some of the species during my first sample of which many might have stayed in the pads for several more days.

### Conclusions

The present study provides quantitative details about the successional patterns of a diverse coprophilous beetle community from Southern Michigan that were sampled throughout the year, including the winter months. The method used to study succession is less time consuming, requires less experimental groups and allows to detect the changing attractiveness of aging dung to newly arriving coprophilous beetles. On the other side, the method is unable to detect for how long dung stays attractive for species that already settled in the dung. In addition, succession should be followed for longer than just 3 d. It would also be helpful to close the massive knowledge gaps about the larval development and trophic preferences of all species in the SC to be able to group them consistently into functional groups.
